# What Does Unexpected Suboptimal Response During Ovarian Stimulation Suggest, an Overlooked Group?

**DOI:** 10.3389/fendo.2021.795254

**Published:** 2021-12-22

**Authors:** Bijun Wang, Wenxia Liu, Yi Liu, Wen Zhang, Chenchen Ren, Yichun Guan

**Affiliations:** ^1^ Reproductive Medicine Center, The Third Affiliated Hospital of Zhengzhou University, Kangfuqian Road, Zhengzhou, China; ^2^ Fuwai Central China Cardiovascular Hospital, Zhengzhou, China; ^3^ Gynecology and Obstetrics, The Third Affiliated Hospital of Zhengzhou University, Zhengzhou, China

**Keywords:** suboptimal ovarian response, IVF, controlled ovarian stimulation, cumulative clinical pregnancy rate, cumulative live birth rate

## Abstract

Unlike poor ovarian response, despite being predicted to be normal responders based on their ovarian reserve markers, many patients respond suboptimally to ovarian stimulation. Although we can improve the number of retrieved oocytes by increasing the recombinant FSH dose and adding LH, the effect of suboptimal ovarian response on cumulative live birth rate (CLBR) and offspring safety is unclear. This study focuses on the unexpected suboptimal response during ovulation induction, and its causes and outcomes are analysed for the first time with a large amount of data used to compare the cumulative pregnancy rate (CPR), CLBR and offspring safety of patients with one complete ART cycle with all embryos used. Our analysis included 5218 patients treated with the GnRH agonist long protocol for their first IVF–embryo transfer (ET) cycles. Patients were divided into two groups according to whether the ovarian response was suboptimal. Propensity score matching (PSM) was utilized for sampling at up to 1:1 nearest-neighbour matching with caliper 0.05 to balance the baseline and improve comparability between the groups. Results showed that age, BMI and basal FSH were independent risk factors for slow response; the initial dosage of Gn, FSH on the first day of Gn, and LH on the first day of Gn were independent protective factors for suboptimal response. Suboptimal responders were also more likely to have irregular menses. Regarding the clinical pregnancy rate of the fresh IVF/ICSI-ET cycles, the adjusted results of the two groups were not significantly different. There was no difference in the CPR, CLBR, or offspring safety-related data, such as gestational age, preterm delivery rate, birthweight, birth-height and Apgar Scores between the two groups after PSM. Age-related changes in the number of oocytes retrieved from women aged 20–40 years old between the two groups were different, indicating that suboptimal response in elderly patients suggests a decline in ovarian reserve. Although we can now improve the outcomes of suboptimal responders, it increases the cost to the patients and the time to live birth, which requires further attention.

## Introduction

In assisted reproductive medicine, the standard gonadotropin-releasing hormone agonist (GnRH-a) downregulation regimen is the most commonly used regimen for controlled ovulation induction in young women with normal gonadotropin releasing hormone (GnRH) levels (1). In this protocol, endogenous hormones are suppressed and recombinant FSH (rFSH) is used to achieve multiple follicular growth (2). Optimization of the ovarian response to controlled ovarian stimulation (COS) in the *in vitro* fertilization (IVF) protocol remains an important topic of debate. Not every woman undergoing COS has the same degree of ovarian response, and some need increased hormone stimulation for follicular development to continue and reach completion (3).

Ovarian resistance to Gn stimulation remains a largely underemphasized issue in the field of reproductive medicine. In COS, there is also a unique type of patient with a slow ovarian response. In this patient, follicle recruitment and hormone levels are normal at the beginning of fixed dose treatment on the first day of the cycle; however, when the same dose was continued, the serum level and follicle size did not significantly increase (4). While there is consensus on the definition of poor ovarian response based on the number of oocytes retrieved following the ovarian stimulation, there is no precise definition of suboptimal ovarian response during ovulation induction in assisted reproductive technology (ART). Previous research has defined suboptimal ovarian response as serum E2 levels <658.8 pmol/L on the sixth to eighth day of stimulation with ultrasound evidence of at least six follicles ranging from 6 to 10 mm but with no follicle with a mean diameter ≥10 mm. Normal response was defined as ultrasound evidence of at least six follicles ranging from 6 to 10 mm with at least one follicle with a mean diameter ≥10 mm (5). According to the literature, 15% of patients show a suboptimal response and require increased or prolonged FSH stimulation to continue follicular growth, which leads to substantial economic losses and time costs. Clinically, suboptimal response is often ignored. If not handled properly, a suboptimal response can easily turn into a poor response and can even result in cycle cancellation (6).

Even patients with a predicted normal response can have a suboptimal response. There have been few studies in which data from complete cycles have been used to compare the live birth rate or cumulative live birth rate (CLBR) (7) of patients with unexpected suboptimal response versus normal response. This study focused on the unexpected suboptimal response during ovulation induction, and its causes and outcomes are analysed, for the first time with a large amount of data used to compare the cumulative pregnancy rate (CPR), CLBR and offspring safety of patients with one complete ART cycle with all embryos used.

## Materials and Methods

### Patients

This retrospective, single-centre, cohort study included ART treatment cycles carried out at the Reproduction Center of the Third Affiliated Hospital of Zhengzhou University between January 2015 and October 2019 in GnRH agonist downregulated stimulation protocols. The study was approved by the institutional review board of the Third Affiliated Hospital of Zhengzhou University.

Among the candidates undergoing a GnRH agonist long protocol for the first-time IVF–embryo transfer (IVF–ET) or intracytoplasmic sperm injection (ICSI) cycles, only those aged 20–40 years with an antral follicle count ≥5-7, AMH≥0.5-1.1mg/L(7.85–10 pmol/L), basal FSH concentration (on day 3 of a spontaneous menstrual cycle) <10 IU/L, hysteroscopic evidence of a normal uterine cavity and no ovarian stimulation over the previous 6 months, presence of both ovaries, and normal karyotypes in both partners were included (8). All patients were treated with a conventional starting Gn dose of 150–225 IU recombinant FSH (rFSH) in a fixed GnRH agonist protocol. The following exclusion criteria were used: body mass index [BMI=weight (kg)/height (m^2^)] <18.0 or BMI>28, polycystic ovarian syndrome (PCOS), endometriosis, chromosomal abnormalities, endocrinological and/or autoimmune disorders, or presence of only one ovary.

### Ovarian Stimulation Protocol (GnRH-Agonist Long Protocol)

All patients underwent a GnRH agonist long protocol with the GnRH agonist triptorelin (Diphereline, 3.75 mg GnRH-a, IPSEN, France) on the second day of the menstrual cycle, and Gn stimulation was initiated after standard downregulation (9). Suboptimal response was defined as serum E2 levels <658.8 pmol/L on the sixth to eighth day of stimulation with ultrasound evidence of at least six follicles ranging from 6 to 10 mm but with no follicle with a mean diameter ≥10 mm. For patients with suboptimal response, follicular stimulation was continued using 75–150 IU of recombinant luteinizing hormone (LH) (Luveris, Serono) in addition to an increasing dose of rFSH, which has been shown to achieve pregnancy and implantation rates similar to those of patients with normal response (10). Normal response was defined as ultrasound evidence of at least six follicles ranging between 6 and 10 mm with at least one follicle with a mean diameter ≥10 mm. If at least three follicles were > 18 mm in diameter, human chorionic gonadotropin (hCG, Merck Serono, Italy) was administered as the trigger. Transvaginal oocyte retrieval was performed 36 h later, and ET was performed on day 3 after oocyte retrieval. For frozen embryo transfers (FETs), embryos that were not suitable for cryopreservation on day 3 were cultured until days 5 or 6 and vitrified if they reached the blastocyst stage. Luteal-phase support with vaginal combined oral progesterone was started three days before FET.

### Outcome Measures of CLBR

The primary outcome was the number of cumulative live births per ovum pick-up (OPU), which was defined as the first live-born baby at ≥28 weeks of gestation resulting from a completed ART cycle, including all fresh ETs and FETs resulting from the associated ovarian stimulation. If a live birth occurred, the patients achieved the outcome regardless of subsequent cycles. According to this definition, multiple deliveries or multiple live births from the same pregnancy were considered one live birth. CLBRs were calculated as the proportion of cycles in which the first live birth was achieved (11).

### Statistical Analysis and Sample Size

Statistical analysis was performed using SPSS software (version 17.0 for Windows; SPSS Inc., Chicago, IL, USA). If continuous variables were normally distributed, they were presented as mean value ± standard deviation (SD). If continuous data were not normally distributed, median [range] were presented. Categorical data are described by number of cases, including numerator/denominator and percentages. A value of p<0.05 was considered significant. Continuous variables are calculated *via* dependent-sample t tests or Mann-Whitney U test, as appropriate. Categorical variables are analysed *via* chi-square or Fisher exact test, as appropriate. A multivariable logistic regression analysis was used to evaluate the relative prognostic significance of age, BMI, AMH, basal FSH, initial dosage of Gn, and total dosage of Gn used in relation to the clinical pregnancy rate and abortion rate. Interactions between independent covariates were adjusted.

PSM was utilized for sampling at up to 1:1 nearest-neighbour matching with caliper (0.05) to balance the baseline and improve the comparability between groups. The PSM allowed each patient who underwent suboptimal response to be matched to normal response patient with similar characteristics, which included age, BMI, AMH, basal FSH, initial dosage of Gn, total dosage of Gn used. A total of 649 participants (after PSM) would provide 95% power to detect no difference in CLBR, assuming a standard deviation of 2 and an alpha of 0.05.

## Results

### The Risks of Suboptimal Response

**Figure 1:**
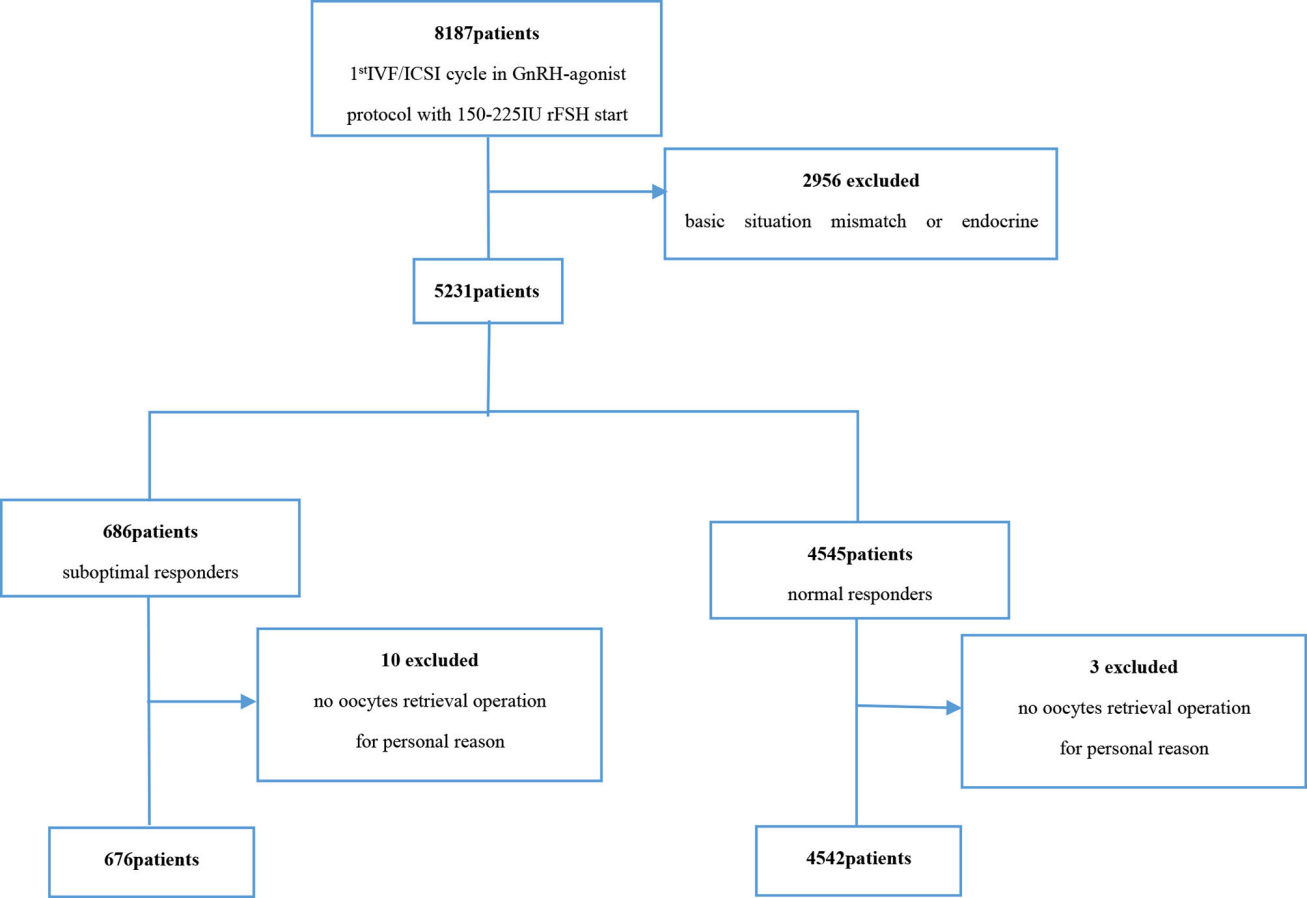
Flowchart of the included population.

Prior to matching, 676 patients with suboptimal response and 4,542 patients with normal response were available for analysis (**Figure 1**). After PSM, a total of 649 patients with suboptimal response were successfully matched to 649 patients with normal response (**Table 1**).

**Table 1:** Comparison of baseline characteristics patients before and after PSM.

	Before PSM			After PSM		
	Suboptimal ovarian response (n=676)	Normal ovarian response (n=4542)	*p*	Suboptimal ovarian response (n=649)	Normal ovarian response (n=649)	*p*
Age (year)	30.00 ± 4.009	29.21 ± 3.889	0.000	29.15 ± 3.921	29 ± 4.201	0.618
BMI (kg/m2)	23.944 ± 2.543	22.900 ± 2.455	0.000	23.032 ± 2.56	23 ± 2.43	0.652
Basal FSH (IU/L)	6.848 ± 1.649	6.457 ± 1.557	0.000	6.81± 1.632	6.9 ± 1.509	0.317
Basal LH(IU/L)	6.071 ± 3.725	5.683 ± 3.326	0.050	6.011 ± 3.692	5.932 ± 3.861	0.706
Basal E2 (pmol/L)	166.272 ± 111.664	177.425 ± 166.942	0.093	168.452 ± 109.264	173.385 ± 145.758	0.490
Basal TSH(IU/L)	2.687 ± 1.589	2.889 ± 3.705	0.162	2.574 ± 1.479	2.395 ± 2.021	0.069
AMH (pmol/L)	21.972 ± 9.87	22 ± 10.10	0.282	21.874 ± 9.89	21.932 ± 9.928	0.918
Oligomenorrhea rate	425/676	2684/4575	0.000	420/649	374/649	0.000
Initial dosage of Gn (IU)	171.34 ± 30.71	190 ± 32.33	0.001	171.55 ± 30.91	170 ± 29.86	0.891
Total dosage of Gn used (IU)	3058.30 ± 1152.53	2300± 961.06	0.001	2966.62 ± 1055.73	3000 ± 1105.62	0.916
Duration of Gn used (d)	16.45 ± 2.710	13.04 ± 1.983	0.000	16.32 ± 2.50	14 ± 2.03	0.000
Cost (RMB)	14253.94 ± 3075.64	10943 ± 2067.94	0.000	13827.088 ± 2817.31	13273.478 ± 1993.05	0.231
Frist day on Gn						
LH	0.751 ± 0.376	0.835 ± 0.541	0.013	0.74 ± 0.38	0.81 ± 0.38	0.022
FSH	2.323 ± 1.774	2.895 ± 1.361	0.000	2.31 ± 1.16	2.7 ± 1.29	0.001
E2	133.861 ± 67.426	145.325 ± 52.652	0.000	151.645 ± 59.329	159.542 ± 66.901	0.025
7or8 days after Gn						
LH	0.494 ± 0.357	0.934 ± 1.212	0.003	0.50 ± 0.36	0.66 ± 0.47	0.009
E2	431.788 ± 212.509	1705.532 ± 1313.424	0.000	483.365 ± 292.033	1642.055 ± 1009.435	0.000
HCG injection day						
LH	1.333 ± 1.445	1.410 ± 1.043	0.092	1.365 ± 1.47	1.447 ± 0.990	0.152
E2	13397.476 ± 6561.658	15418.815 ± 7206.041	0.979	14593 ± 8253.094	15021 ± 8022.038	0.883
P	4.493 ± 2.097	4.438 ± 2.378	0.853	4.391 ± 1.884	4.469 ± 2.774	0.297

Gn, gonadotropin.

BMI, Body mass index.

**Table 1** presents the patient characteristics before and after matching. There were no differences in basal LH, basal E2, AMH or TSH between the two groups, but the age, BMI and basal FSH of the unexpected suboptimal response group were higher (**Table 1**). Compared with the normal response group, initial dosage of Gn was lower (171.34 ± 30.71, 190 ± 32.33, p=0.001), and the total Gn dose (3,058.30 ± 1,152.53 vs. 2300± 961.06, p=0.001) and days of ovulation (16.45 ± 2.710 vs. 13.04± 1.983, p=0.000) were higher in the unexpected suboptimal response group (**Table 1**). After PSM, there were no difference in age, BMI and basal FSH, initial dosage of Gn and total dosage of Gn used.

**Table 2** presents Multivariate logistic regression analysis of slow ovarian response, and it showed that age, BMI and basal FSH were independent risk factors for slow response and the initial dosage of Gn, FSH on the first day of Gn, and LH on the first day of Gn were independent protective factors for suboptimal response.

**Table 2:** Multivariable logistic regression of suboptimal ovarian response (before PSM).

Parameter	β	*P*
Age	0.039	0.029
BMI	0.142	0.000
Basal FSH (IU/L)	0.302	0.000
Initial dosage of Gn (IU)	-0.101	0.000
FSH on first day of Gn	-0.384	0.000
LH on first day of Gn	-0.488	0.009

### The Difference of Serum LH Levels

**Figure 2:**
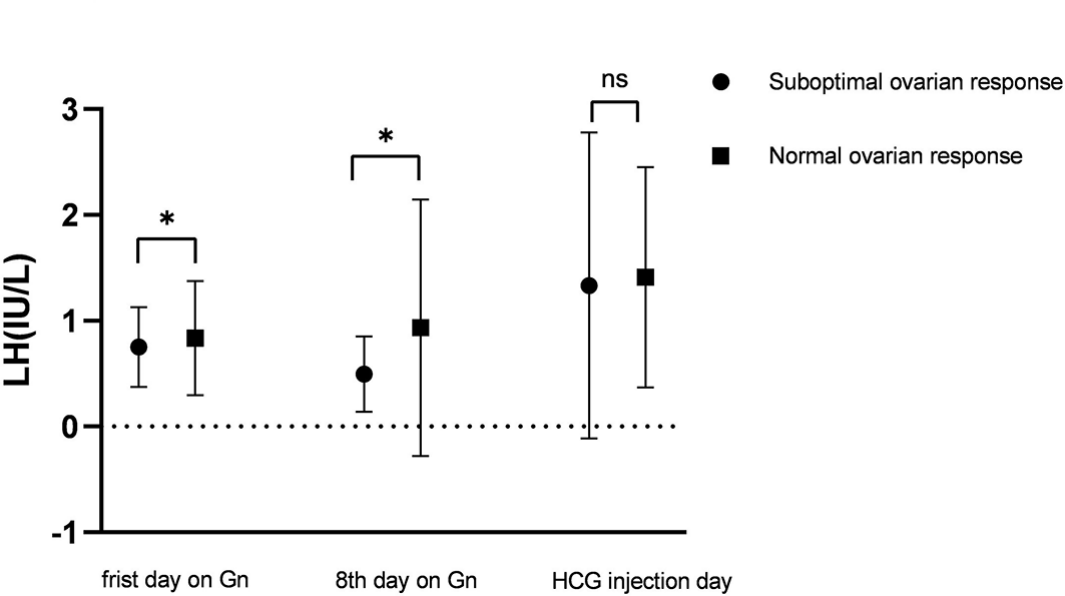
LH levels on the first day of Gn, the 8th day of Gn and the day of hCG injection in the suboptimal response group and normal response group. *means P < 0.05, NS means no statistical significance.

LH, FSH and E2 levels on the first day and LH and E2 levels on the 8th day of Gn in the suboptimal response group were significantly lower than those in the normal response group (**Table 1**). But on the day of hCG injection, there was no significant difference in the LH level between the two groups (**Figure 2**).

### About IVF/ICSI Outcomes

The number of oocytes retrieved in the fresh cycles was significantly higher in the normal ovarian response group (13.41 ± 6.629 vs. 15.36± 6.658, p=0.000), but other results such as the good-quality embryo rate, the blastocyst rate, the pregnancy rate, or the early abortion rate showed no difference (**Table 3**). And all of these showed no difference after PSM. Regarding CPR in the fresh IVF/ICSI cycles, the adjusted results did not show any significant difference (**Table 4**). Regarding the PSM results, there was no difference in CPR, CLBR, or offspring safety related data such as gestational age, preterm delivery rate, birthweight, birth-height and Apgar Scores between the two groups (**Table 5**). However, a suboptimal response increases increased the cost and the time to live birth (**Tables 1** and **5**).

**Table 3:** Comparison of laboratory index and IVF/ICSI outcomes of patients before and after PSM.

	Before PSM			After PSM			
	Suboptimal ovarian response (n=676)	Normal ovarian response (n=4542)	*p*	Suboptimal ovarian response (n=649)		Normal ovarian response (n=649)	*p*
Number of oocytes retrieved	13.411 ± 6.629	15.36 ± 6.658	0.000	14.052 ± 6.633		15.004 ± 6.28	0.884
Number of available embryos	7.368 ± 3.591	7.708 ± 4.723	0.081	7.434 ± 4.707		7.203 ± 4.421	0.427
Number of good-quality embryos	4.259 ± 3.665	4.441 ± 3.798	0.242	4.284 ± 3.671		4.245 ± 3.439	0.591
Good-quality embryo rate	58.9% (1844/3133)	57.6% (11974/20787)	0.185	58.4% (1826/3126)		58.5% (1893/3234)	0.922
Number of blastocysts	3.552 ± 3.450	3.856 ± 3.591	0.039	3.613 ± 3.472		3.446 ± 3.373	0.284
Blastocyst rate	63.1% (2338/3707)	63.5% (17153/27004)	0.593	62.9% (2293/3641)		62.6% (2289/3655)	0.757
Average number of embryos implanted	1.69 ± 0.462	1.60 ± 0.490	0.952	1.68 ± 0.438		1.65 ± 0.471	0.235
Cancellation because of P rise	39.5% (79/200)	35.7% (530/1485)	0.292	39.5% (75/185)		35.7% (75/181)	0.862
Clinical pregnancy rate	67.2% (314/476)	60.8% (1918/3057)	0.484	67.2% (312/464)		67.3% (315/468)	0.983
Abortion rate	11.3% (36/314)	11.8% (221/1918)	0.871	11.5% (36/312)		11.7% (37/315)	0.935
Live birth rate	57.7% (275/476)	55.3% (1692/3057)	0.322	58.8% (273/464)		57.9% (271/468)	0.901

**Table 4:** Logistic and multivariate regression analysis before PSM (adjusted for age, BMI, AMH, basal FSH, initial dosage of Gn, total dosage of Gn used).

Parameter	Suboptimal ovarian response (n=676)	Normal ovarian response (n=4542)	β	OR	*P*
Clinical pregnancy	62.5% (293/469)	60.8% (1813/2983)	-0.105	0.900	0.266
Abortion rate	12.3% (36/292)	12.5% (226/1812)	-0.115	0.968	0.553

**Table 5:** Stratified analysis of CLBR after PSM (adjusted for age, BMI, AMH, basal FSH, initial dosage of Gn, total dosage of Gn used).

Parameter	Suboptimal ovarian response (n=649)	Normal ovarian response (n=649)	*P*
Cumulative pregnancy rate	83.5% (542/649)	84.7% (550/649)	0.550
Cumulative live birth rate	78.4% (509/649)	79.9% (519/649)	0.461
Time to Live Birth(days)	525 ± 181.275	471 ± 193.781	0.000
Gestational age (weeks)	38.98 ± 1.8	39.11 ± 1.8	0.184
Preterm delivery, <37weeks	5.14% (27/525)	5.39% (28/519)	0.612
Birthweight(g)	3464.73 ± 499.86	3501.66 ± 523.01	0.200
Birth-height(cm)	50.11 ± 2.09	50.19 ± 2.46	0.519
1-minute Apgar Scores	10 ± 0.3	10 ± 0.2	1.000
1-minute Apgar Scores	10 ± 0.3	10 ± 0.3	1.000

**Figure 3:**
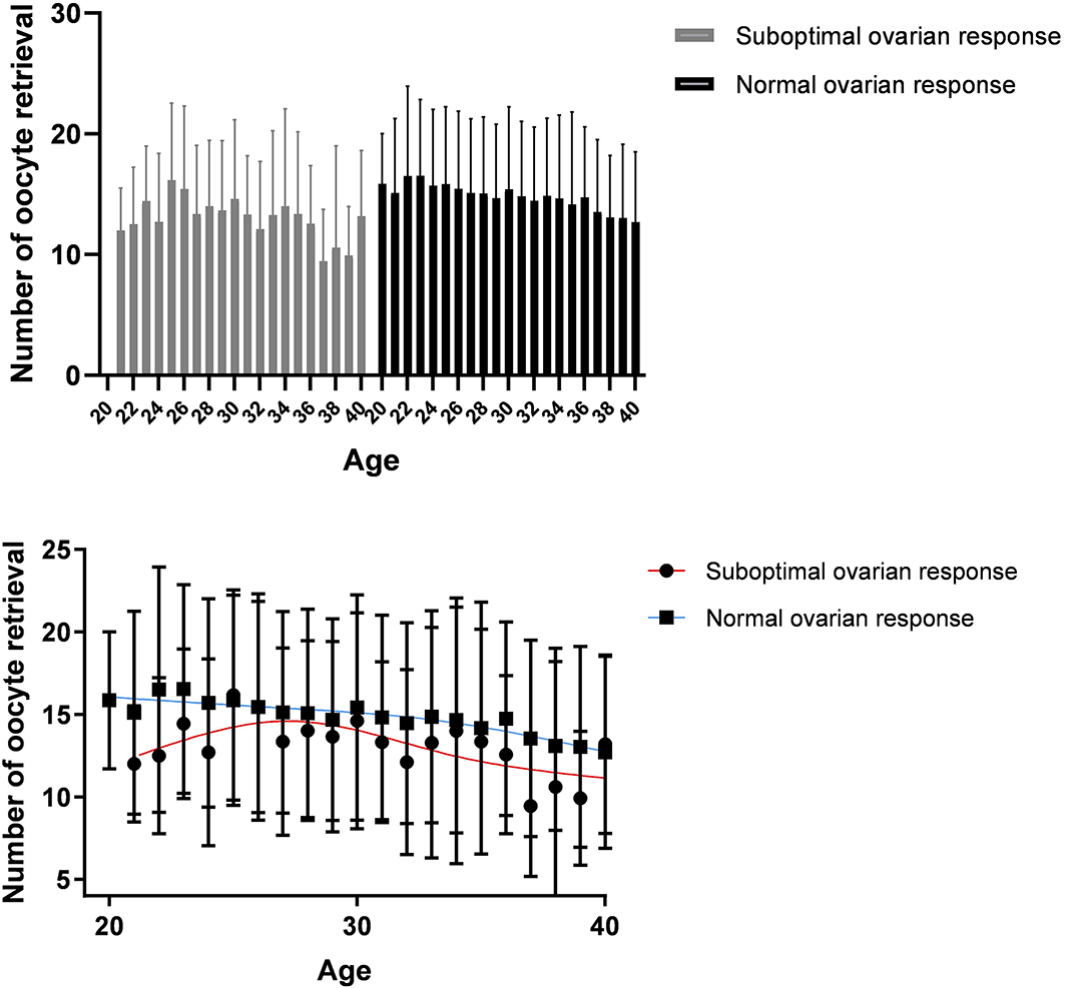
Age-related changes in the treatment outcomes of women aged 20–40 years in the normal ovarian response group and suboptimal ovarian response group.

**Figure 3** shows the trend of the number of oocytes retrieved with age. Age-related changes in the number of oocytes retrieved from women aged 20–40 years between the two groups were different (**Figure 3**). When the patient age was above than 28 years, the number of oocytes retrieved declined at different rates.

## Discussion

An appropriate ovarian response to controlled ovarian hyperstimulation (COH) is extremely important for the success of IVF (12, 13). Previous studies have suggested that obesity, advanced age, LH deficiency and decreased ovarian reserve are risk factors for slow ovarian response (14). However, a specific subgroup of patients who are supposed to be normal responders based on their ovarian reserve may respond suboptimally, manifesting as an “unexpected suboptimal response” during COH.

There is consensus that age, BMI and AMH directly influence the Gn dose and the pregnancy outcome of IVF (15, 16). During the process of FSH-mediated stimulation of follicular growth, the serum FSH concentration needs to pass the “threshold” to initiate multi-follicular development. This “threshold” effect is weakened in obese patients, resulting in the need for additional doses of FSH to stimulate follicular growth. Therefore, in this study, older women, obese women and those with a low ovarian reserve were excluded, and the population with a normal ovarian reserve and the expectation of a normal ovarian response were specifically selected to explore the risk factors for unexpected suboptimal response. Our results showed that there were no differences in basal endocrine and metabolic levels between the two groups, but the age, BMI, and basal FSH of the unexpected suboptimal response group were still higher. The multivariate logistic regression analysis of slow ovarian response showed that age, BMI and basal FSH were independent risk factors for slow response. Interestingly, we found that the oligomenorrhea rate was significantly higher in the unexpected suboptimal response group.

Treatment with Gn agonists is a current method used for infertility treatment. Gn agonists can significantly suppress pituitary function, but in some patients, this will result in excessive suppression, which significantly reduces LH levels and induces a slow ovarian response (17). Our research shows that LH, FSH and E2 levels on the first day and LH and E2 levels on the 8th day of Gn treatment were significantly lower in the suboptimal response group than in the normal response group. On the day of hCG injection, there was no significant difference in the LH level between the two groups. The issue of LH supplementation in ART cycles has been a matter of debate for years, but it still needs to be clarified. Some hold that LH has no role or might even negatively impact the IVF cycle by inducing early luteinization (18), while others hold that administration of 75–150 IU of LH does not induce early luteinization (19, 20). Regarding the suboptimal response, we continued follicular stimulation using 75–150 IU of recombinant LH in addition to increasing doses of rFSH. But our results showed that the ET cancellation rate due to increased progesterone was identical in the suboptimal response group as that in the normal response group, without inducing early luteinization.

Our study also suggested that the initial dose of Gn in the suboptimal ovarian response group was lower than that in the normal group. Some studies suggest that this is the main cause of suboptimal response in patients for whom a normal response would otherwise be expected with a long-agonist regimen (21). After controlling for confounding endocrine disorders and ovarian reserve, identifying the factors that cause a suboptimal response is often a challenge for a clinician when counselling patients. A suboptimal response means higher total Gn, a longer ovulation time and a higher cost. A previous retrospective cohort study demonstrated that an increase in the average daily dose of Gn was the only variable significantly associated with a higher oocyte yield in women with normal ovarian reserve undergoing two IVF cycles (22). Daily dosages of FSH in the range of 150–225 IU create maximal stimulation in patients with low, normal and high responders, Increasing FSH to a dosage of 300 IU will not create more oocytes (23, 24). Poor response is indisputably recognized as problematic, as the far below-average prognosis for a live birth, especially among older women, sheds doubt on the added value of ART (25), where in producing oocyte numbers in the range of 4–9 is optimal. However, unlike these populations, the pregnancy outcome of patients with a suboptimal response can be made to be the same as that of the normal population by increasing the dose of rFSH and LH (19). Our study also suggested that even through the number of oocytes retrieved in the fresh cycles was significantly higher in the normal ovarian response group than in the suboptimal ovarian response group, there was no difference in the good-quality embryo rate, the blastocyst rate, the pregnancy rate, or the early abortion rate. Multivariate logistic regression analysis showed that suboptimal ovarian response did not affect clinical pregnancy rate and abortion rate adjusted for age, BMI, AMH, basal FSH, initial dosage of Gn, and total dosage of Gn used.

To exclude the effects of age on ovarian reactivity, the number of oocytes retrieved was calculated for different age subgroups within the two groups. Results showed that the age-related changes in the number of oocytes retrieved from women aged 20–40 years between the two groups were different. Specifically, above 28 years of age, the number of oocytes retrieved declined at different rates between the two groups. This suggests that with increasing age, among older women, patients with normal ovarian response had a significant advantage in terms of their ovarian reserve over patients with suboptimal ovarian response. Does this suggest that ovarian reserve declines more quickly in patients with suboptimal ovarian response?

The primary goal of ART is to provide effective and safe personalized solutions to help infertile couples achieve a live birth. This objective should be attained with the mindset of securing the shortest time to live birth while avoiding negative consequences for the mother and new-born. One of the major strengths of our retrospective longitudinal study is that after PSM, we had a large homogeneous group of women who had the same age, BMI, and AMH level and the same initial dose of Gn. With the PSM results, there was no difference in the CPR or CLBR between the two groups. This suggests that the occurrence of a suboptimal ovarian response increases only the time of ovulation promotion but does not affect the outcome in terms of pregnancy achievement. To investigate suboptimal response affects offspring safety, we compared relevant data. There was no difference in gestational age, preterm delivery rate, birthweight, birth-height and Apgar Scores between the two groups after PSM. But suboptimal response still increases the cost to the patient and the time to live birth.

However, caution is needed owing to limitations that do exist and need to be highlighted. First, the retrospective study design is per its definition associated with inherent biases that may affect our results. Finally, although the number of oocytes was found to increase in the normal response group compared with the unexpected suboptimal response group, our design could not allow for evaluation of the effect on cumulative live birth rates from fresh ETs. Although we can now improve the outcomes of suboptimal responders, it increases the cost to the patient and the time to live birth. Especially in older women, does suboptimal response suggest faster decline in ovarian function? These still requires us to explore the causes in different populations.

## Data Availability Statement

The raw data supporting the conclusions of this article will be made available by the authors, without undue reservation.

## Author Contributions

BW and WL designed the study and wrote the manuscript. CR and YG designed the study. YL and WZ analysed the data. All authors contributed to the article and approved the submitted version.

## Funding

This work was supported by grant from Joint Project of Medical Disciplines of Henan Province (LHGJ20200428, BW; LHGJ20200450, WL).

## Conflict of Interest

The authors declare that the research was conducted in the absence of any commercial or financial relationships that could be construed as a potential conflict of interest.

## Publisher’s Note

All claims expressed in this article are solely those of the authors and do not necessarily represent those of their affiliated organizations, or those of the publisher, the editors and the reviewers. Any product that may be evaluated in this article, or claim that may be made by its manufacturer, is not guaranteed or endorsed by the publisher.
